# Characterization of the arylsulfatase I (*ARSI*) gene preferentially expressed in the human retinal pigment epithelium cell line ARPE-19

**Published:** 2009-03-06

**Authors:** Mio Oshikawa, Ron Usami, Seishi Kato

**Affiliations:** 1 Department of Rehabilitation Engineering, Research Institute, National Rehabilitation Center for Persons with Disabilities, Saitama, Japan; 2 Department of Applied Chemistry, Graduate School of Engineering, Toyo University, Saitama, Japan

## Abstract

**Purpose:**

The aim of this study was to characterize the arylsulfatase I (*ARSI*) gene that has been shown to be preferentially expressed in the human retinal pigment epithelium cell line ARPE-19 and to propose it as a candidate gene responsible for inherited eye diseases such as retinitis pigmentosa (RP).

**Methods:**

Full-length cDNA clones encoding *ARSI*, arylsulfatase A (*ARSA*), and sulfatase modifying factor 1 (*SUMF1*) were isolated from ARPE-19 cDNA libraries constructed using the vector-capping method. The expression vectors for their FLAG-tagged proteins were transfected into ARPE-19 cells, and the expression products were characterized by western blot analysis and arylsulfatase assay. The entire region of the *ARSI* gene locus was sequenced using the genomic DNA samples of 68 RP patients.

**Results:**

Transiently produced ARSI-FLAG was localized to the endoplasmic reticulum and was detected in the cellular fraction and the medium. When *ARSI-FLAG* and *SUMF1-FLAG* were coexpressed, the conditioned medium of the transfected cells showed arylsulfatase activity at a range of neutral pH. No mutation was found in the *ARSI* gene locus of the RP patients examined.

**Conclusions:**

ARSI may be a secreted sulfatase and may function in the extracellular space. Although *ARSI* may not be a causative gene for lysosomal storage diseases, preferentially expressed in the eye, *ARSI* would be a candidate gene causing inherited eye diseases for future mutation screening.

## Introduction

Sulfatases represent a large enzyme family that hydrolyzes sulfate esters and sulfamates [1]. The majority of human sulfatases reside in lysosomes and are responsible for the degradation of sulfated carbohydrates. As their enzymatic activities have been measured using small aryl substrates such as p-nitrophenol sulfate or 4-methylumbelliferyl sulfate (4-MUS), these sulfatases are named arylsulfatase (ARS). Alignment of the amino acid sequences of ARSs has shown the presence of two conserved regions at their N-terminal end. Signature sequence I contains a cysteine residue that is converted into the catalytically active formylglycine (FGly) residue [2] by post-translational modification with sulfatase modifying factor 1 (SUMF1) [3,4] in the endoplasmic reticulum (ER). The search analysis for sulfatase genes across the human genome using the above conserved sequences has revealed a total of 17 sulfatase genes, including 4 novel genes, arylsulfatase H (*ARSH*), arylsulfatase I (*ARSI*), arylsulfatase J (*ARSJ*), and arylsulfatase K (*ARSK*) [5]. *ARSI* and *ARSJ* are regarded as *ARSB* paralogs. Recently, *ARSI* and *ARSJ* cDNAs have been cloned and expressed in HeLa cells, but no ARS activity has been detected [6].

Mutations or deficiencies of the *ARS* genes have been found to cause lysosomal storage diseases such as mucopolysaccharidoses (MPS) and metachromatic leukodystrophy [7]. Patients with these diseases often suffer from retinal degeneration similar to that seen in retinitis pigmentosa (RP) [8-13]. The lysosomal storage in retinal pigment epithelium (RPE) cells is suspected to cause the degeneration of photoreceptor cells [11].

RP is a set of hereditary retinal diseases caused by degeneration of photoreceptor cells. More than 45 genes responsible for RP have been identified and account for approximately 60% of RP patients in the United States [14]. However, many of the responsible genes remain unidentified. To identify novel candidate genes causing RP, we have been searching for genes preferentially expressed in human retina-derived cell lines [15,16]. During this search, we found that *ARSI* is preferentially expressed in RPE cell line ARPE-19. We thought that *ARSI* might be a possible candidate for an RP-responsible gene because mutations of ARSs are known to cause MPS accompanied by symptoms of RP. In this paper, we describe the cloning of full-length cDNAs of ARS-related genes, the transient expression of *ARSI* cDNA and the detection of ARS activity of its product. Further, we report the results of mutation screening of the *ARSI* gene locus in Japanese RP patients.

## Methods

### cDNA cloning

The cloning of full-length cDNAs was performed using a full-length cDNA collection consisting of 93,504 clones randomly isolated from the cDNA libraries that had been constructed from total RNA of ARPE-19 cells using a vector-capping method as described in the previous papers [15,16]. Full-length cDNA encoding *ARSI* was identified by the 5′-end single-pass sequencing of 24,000 clones. Those for *ARSA* and *SUMF1* were obtained by polymerase chain reaction (PCR)-based screening of the remaining 69,504 clones. Full sequencing of the obtained clones was performed using a primer walking method, and the determined sequences were registered to DDBJ/GenBank/EMBL under accession numbers, AB448735 (*ARSI*), AB448736 (*ARSA*), and AB448737 (*SUMF1*).

### Sequencing

Sequencing reactions were performed with a BigDye^®^ Terminator Cycle Sequencing Kit (Applied Biosystems, Foster City, CA). The M13 universal forward primer was used for the 5′-end single-pass sequencing. Primers used for genome sequencing are listed in Table 1. Sequencing reaction products were run on an automated capillary sequencer (Applied Biosystems 3130xl Genetic Analyzer, Applied Biosystems).

**Table 1:** Primers used for sequencing the arylsulfatase I gene locus.

**Primer**	**Position**	**Sequence (5′->3′)**
**Forward**		
SF01	−232	CCCGGTTGGACTAGGAAATGATGGGCTT
SF02	229	GGAAGAGGGGAGTCAGAGGATG
SF03	710	CCCCAGCCTCCCCACATCATCT
SF04	1140	CATCTGGTCATCCCTTTGTTAG
SF05	1588	GGCGAGGCAGCAGGATTACAGG
SF06	2041	GGGGTGGTGGTGCAGGTTAGTG
SF07	2393	TTGGTGACTCTTAGCTGGC
SF08	2887	CTGGAGGTGAGAAAAAGGTGTGGG
SF09	3313	TCTGTTCCTGACCACCGAGCGA
SF10	3745	TGCTCTAGGCCCCAGTGCAAGTTA
SF11	4143	CCAGTGATTTTGAGGATTCGATGAG
SF12	4525	CACCTTCCTGGGCTCGCTCACG
SF13	4981	TTATTGGGAAGGTGGCGTGCGG
SF14	5377	GTGGAACCTGGAACGAATGGCC
SF15	5836	CAGGGAGTTGGAGGGTGTAGAG
**Reverse**		
SR01	250	CATCCTCTGACTCCCCTCTTCC
SR02	731	AGATGATGTGGGGAGGCTGGGG
SR03	907	CTTGAGCCACGCCTACCTGCC
SR04	1161	CTAACAAAGGGATGACCAGATG
SR05	1609	CCTGTAATCCTGCTGCCTCGCC
SR06	2062	CACTAACCTGCACCACCACCCC
SR07	2472	CAACTGGGCCGGTCTTCATAG
SR08	2910	CCCACACCTTTTTCTCACCTCCAG
SR09	3334	TCGCTCGGTGGTCAGGAACAGA
SR10	3768	TAACTTGCACTGGGGCCTAGAGCA
SR11	4167	CTCATCGAATCCTCAAAATCACTGG
SR12	4546	CGTGAGCGAGCCCAGGAAGGTG
SR13	5002	CCGCACGCCACCTTCCCAATAA
SR14	5398	GGCCATTCGTTCCAGGTTCCAC
SR15	5857	CTCTACACCCTCCAACTCCCTG
SR16	6262	TCAACCCCCCCGACCCCTGCCC
SR17	6442	GTCCTGGTACTGGTTTGGGGGCTCTTCACAG

Position 1 corresponds to the nucleotide A of the initiation codon of the ARSI gene.

### Cell culture

Human retinal pigment epithelium cell line ARPE-19, human retinoblastoma cell line Y79, and human embryonal carcinoma cell line NTERA-2 cl.D1 (NT2/D1) were obtained from American Type Culture Collection (Manassas, VA). Human fibrosarcoma cell line HT-1080 and human adenocarcinoma cell line HeLa S3 (sc) were obtained from the Japanese Collection of Research Bioresources (Osaka, Japan). The culture medium was a 1:1 mixture of Dulbecco’s modified Eagle’s medium (DMEM) and Ham’s F-12 medium (Invitrogen, Carlsbad, CA) containing 10% fetal bovine serum (FBS, JRH Biosciences, Lenexa, KS) for ARPE-19, and RPMI1640 medium (Invitrogen) containing 20% FBS for Y79. The other cell lines were cultured in DMEM with 4.5 g/l of glucose (Sigma Aldrich, St. Louis, MO) containing 10% FBS, and for HT-1080 further supplemented with 10 mM HEPES and 0.1 mM MEM nonessential amino acids (Sigma Aldrich). The incubation was performed at 37 °C in a humidified atmosphere of 5% CO_2_ and 95% air (10% CO_2_ and 90% air for NT2/D1).

### Reverse transcription polymerase chain reaction

Total RNA was isolated using Isogen^®^ (Nippon Gene, Tokyo, Japan). The first-strand cDNA was synthesized from total RNA using oligo (dT)_30_ as a primer with SuperScript^TM^ III reverse transcriptase (Invitrogen), and then purified by a Wizard^®^ PCR preps DNA Purification System (Promega, Madison, WI). The cDNA sample equivalent to 100 ng of total RNA was amplified with TaKaRa Ex Taq^TM^ polymerase (Takara Bio Inc., Shiga, Japan). Table 2 shows the primers used for PCR amplification of each cDNA. The PCR conditions were as follows: initial denaturation at 96 °C for 2 min, 40 cycles of heating at 96 °C (98 °C for *ARSI*) for 30 s, annealing at 60 °C (63 °C for *ARSI*) for 30 s, and extension at 72 °C for 2 min (4 min for *ARSI*), followed by final extension at 72 °C for 10 min.

**Table 2:** Primers for reverse transcription polymerase chain reaction and product sizes.

**Gene**	**Primer name**	**Sequence (5′-3′)**	**Product size (bp)**
*EEF1A1*	107fw	CGTAGATTCGGGCAAGTCCACCAC	1265
	1371rv	CACCCACCGCAACTGTCTGTCTCA	
*ARSA*	807fw	TCTAGGCATCCCGTACTC	1047
	1853rv	CAGCCAGGATGACAGCAGA	
*ARSI*	187fw	AGGAGAGAGAAATTAGGAGGCGGG	2800
	2986rv	GTACTGGTTTGGGGGCTCTTCACA	
*SUMF1*	232fw	ATACTCGCGGGAGGCTAA	1324
	1555rv	ACACCCAGAGGTCATGCT	

### In silico expression profile

The counts of the expressed sequence tag (EST) obtained from various tissues were retrieved from the UniGene website. The UniGene cluster number is Hs.591252 for *ARSI*, Hs.88251 for *ARSA*, Hs.149103 for *ARSB*, and Hs.350475 for *SUMF1*.

### Construction of expression vectors

FLAG-epitope-tagged expression vectors for *ARSI*, *ARSA,* and *SUMF1* (*ARSI-FLAG*, *ARSA-FLAG*, and *SUMF1-FLAG*) and a myc-tagged vector for *SUMF1* (*SUMF1-myc*) were constructed. The coding region of each cDNA was amplified using the primers shown in Table 3 and was cloned into pFLAG-CMV-5 (Sigma Aldrich) or pcDNA3.1/myc-His (Invitrogen). ARSICΔ46 lacks the 46 C-terminal amino acids of ARSI. By replacing the signal peptide of ARSI with that of SUMF1, *S1ARSI-myc* was constructed, and the amplified *S1ARSICΔ46* fragment was then subcloned into pFLAG-CMV-5 to construct the expression vector of *S1ARSICΔ46-FLAG*.

**Table 3:** Primers for construction of expression vectors.

**Gene**	**Primer name**	**Sequence (5′-3′)**
*ARSA-FLAG*	370fw-HindIII	taaagctt**ATG**TCCATGGGGGCACCGCG
	1896rv-KpnI	atggtaccGGCATGGGGATCTGGGCAAT
*ARSI-FLAG*	580fw-EcoRI	ctgaattccacc**ATG**CACACCCTCACTGGCTTC
	2287rv-BglII	ttagatctccGATCCGTTGGGACATTAGCCT
*ARSICΔ46-FLAG*	580fw-EcoRI	ctgaattccacc**ATG**CACACCCTCACTGGCTTC
	2211rv-BglII	ttagatctGCCCAGGGCCCCCAAGCA
*SUMF1-FLAG*	15fw-HindIII	tcaagcttCGCGGGACAAC**ATG**GCTG
	1147rv-KpnI	ttggtaccGTCCATGGTGGGCAGGCG
*SUMF1-myc*	20fw-HindIII	ataagcttGACAAC**ATG**GCTGCGCCC
	1147rv-SacII	ttccgcggGTCCATGGTGGGCAGGCG
*S1ARSI-myc*	SUMF1fw	CGCCTGCCCACCATGGAC
	SUMF1rv-XbaI	cttctagaCCCTGCCGCTCCACACAG
	ARSIfw-XbaI	cctctagaCCTCCCCACATCATCTTCA
	ARSIrv-SacII	atccgcggGATCCGTTGGGACATTAGCCT
*S1ARSICΔ46-FLAG*	SUMF1–20fw-EcoRI	atgaattcGACAAC**ATG**GCTGCGCCC
	ARSI-2211rv-BglII	ttagatctGCCCAGGGCCCCCAAGCA

In the "Sequence" column, uppercase letters represent a cDNA sequence and lowercase letters denote a restriction site. Bold underlining indicates an initiation codon. The coding region of each gene was amplified using the primer set. The amplified product was cut with the corresponding restriction enzyme and was then cloned into pFLAG-CMV-5 or pcDNA3.1/myc-His.

### Site-directed mutagenesis

A single amino acid substitution of the *ARSI* gene was performed using the QuikChange^®^ II Site-Directed Mutagenesis Kit (Stratagene, La Jolla, CA) according to the manufacturer’s protocol. A cysteine at position 93, a putative active site of ARSI, was replaced by a serine. The mutation was introduced into *ARSI-FLAG* using an oligonucleotide, 5′-CAT CCA GCC CAT CTC CAC GCC TTC GC-3′, to construct an expression vector of *ARSI-C93S-FLAG*.

### Transfection

Transfection of the expression vector into ARPE-19 cells was performed using the Amaxa Nucleofector^®^ system (Amaxa Inc., Gaithersburg, MD) according to the manufacturer’s directions. The cultured ARPE-19 cells were trypsinized and harvested by centrifugation. The cell pellet was resuspended in Nucleofector^®^ Solution R to a final concentration of 1.5x10^6^ cells/100 μl. When the multiple expression vectors were coexpressed, the total amount of the introduced plasmid DNA vectors was adjusted to be constant by adding a control vector pFLAG-CMV-5a. The cell suspension was mixed with a total amount of 6–10 μg of plasmid DNA, and electroporation was then performed using the program P-20 of the Nucleofector^®^ device (Amaxa). The transfected cells were seeded in the growth medium containing FBS and cultured for 24–72 h. For the enzymatic assay, the medium was replaced with serum-free medium 2 h after transfection.

### Immunofluorescence

The transfected cells were grown on a culture slide for 24 h. The cells were fixed in 4% paraformaldehyde for 15 min and then permeabilized with methanol. FLAG-tagged protein was detected by immunostaining with 20 μg/ml primary anti-FLAG M2 antibody (Sigma Aldrich) and 1:200 secondary anti-mouse IgG Alexa Fluor^®^ 568 antibody (Molecular Probes, Eugene, OR). Endogenous lysosomal and ER proteins were detected with 1:100 primary anti-cathepsin D (H-75) and 1:100 anti-PDI (protein disulfide isomerase, H-160) antibodies (Santa Cruz Biotechnology, Santa Cruz, CA), respectively, and 1:200 secondary anti-rabbit IgG Alexa Fluor^®^ 488 antibody (Molecular Probes). The cells were incubated with primary antibodies at 4 °C overnight and secondary antibodies at room temperature for 4 h in the dark after blocking with 1% BSA (Sigma Aldrich) in PBS for 1 h. The cells were mounted in ProLong^®^ Gold antifade reagent (Invitrogen) and observed using a Bio-Rad MRC-1024 confocal laser microscope (BioRad, Hercules, CA) .

### Preparation of expression products

The ARPE-19 cells transfected with the FLAG-tagged expression vectors were seeded in 6 cm dishes and incubated at 37 °C for 24–72 h. The medium was concentrated on an Ultrafree filter unit (5000NMWL; Millipore, Bedford, MA) and then dissolved in 100 μl of 50 mM Tris-HCl buffer (pH 7.4) containing 0.5% TritonX-100 and 1 mM phenylmethylsulfonyl fluoride (Sigma Aldrich). Trichloroacetic acid (TCA) precipitation was performed by mixing the medium with an equal volume of a 20% (w/v) TCA/acetone solution and was incubated at −20 °C for 3 h. The precipitate was collected by centrifugation and then washed three times with cold acetone. The cells were disrupted by sonication in 50 mM Tris-HCl buffer (pH 7.4) containing 1% Tween-20 and 1 mM phenylmethylsulfonyl fluoride, and were agitated at 4 °C overnight. The soluble cellular extract was separated from the insoluble cellular fraction by centrifugation. The protein concentration of the cellular extract was determined using a DC Protein Assay kit (Bio-Rad).

### Western blot analysis

The soluble cellular extract, the insoluble cellular fraction, the concentrated medium, and the TCA precipitate were treated with an SDS–PAGE sample buffer that contained 50 mM Tris-HCl, pH 6.8, 2% SDS, 6% 2-mercaptoethanol, and 10% glycerol. The mixture was then boiled for 10 min. The samples were electrophoresed on a 10% polyacrylamide gel in a glycine-SDS buffer and transferred onto Immun-Blot PVDF membranes (Bio-Rad). The membranes were blocked with 1% (w/v) nonfat dried milk (Roche Applied Science, Indianapolis, IN) in maleic acid buffer (pH 7.5) for 1 h at room temperature, and then exposed to 10 μg/ml primary anti-FLAG BioM2 antibody (Sigma Aldrich) for 1 h, followed by 1:1,000 alkaline phosphatase-linked anti-biotin antibody (Vector Laboratories, Burlingame, CA) for 30 min. The bands were visualized by nitroblue tetrazolium/5-bromo-4-chloro-3-indolyl-phosphate chromogen (Roche Applied Science). The production level was estimated by measuring the density of the band using a bio-imaging system (ChemiStage CC-16, Kurabo, Osaka, Japan).

### Arylsulfatase assay

The ARS activity of the cellular extract and medium was determined by incubating 20 μl of the sample solution with 80 μl of 6.25 mM 4-MUS (Sigma Aldrich) as a substrate and 0.125 M sodium acetate (pH 5.0–6.0) or Tris-HCl buffer (pH 6.8–9.0) according to the previous report [4]. For the ARSA assay, 0.625 mM 4-MUS was used. Thus, the final concentration of 4-MUS in an assay solution was 5 mM for ARSI and 0.5 mM for ARSA. The enzymatic reaction was performed at 37 °C for 5 h and stopped by adding 1.9 ml of 133 mM glycine-83 mM carbonate buffer (pH 10.7). Fluorescence was read at an excitation of 365 nm and an emission of 445 nm.

### Mutation screening

The mutation screening of the *ARSI* gene locus was performed using the same genome DNA sets isolated from 68 unrelated RP patients as those used for the mutation screening of rhodopsin gene [17]. The study was approved by the Human Genome-Ethics Committee of the National Rehabilitation Center for Persons with Disabilities and was performed in accordance with the Declaration of Helsinki. Informed consent was obtained from all subjects involved in this study.

Genomic DNA was isolated from venous blood using a Puregene^®^ DNA purification kit (Gentra Systems, Minneapolis, MN). A sample for DNA sequencing was prepared by PCR using genomic DNA as a template. The genomic sequence of the *ARSI* locus (NT_029289.10) was retrieved from the NCBI database (Build 36.3). Nucleotide A of the initiation codon of *ARSI* was defined as Position 1. The primer sequences for PCR amplification are listed in Table 4. The first PCR amplification was performed to obtain an amplicon spanning an entire region of the *ARSI* locus. Then 200 ng of genomic DNA were amplified with TaKaRa Ex Taq^TM^ polymerase (Takara Bio) in a 50 μl reaction volume for 30 cycles under conditions of heating for 30 s at 96 °C, annealing for 30 s at 63 °C, and extension for 4 min at 72 °C. The second PCR amplification using the amplicon A1 as a template produced three amplicons (A2, A3, A4). PCR products were sequenced bidirectionally, using the primers listed in Table 1.

**Table 4:** Polymerase chain reaction primers used for amplifying the arylsulfatase I gene locus.

**Amplicon**	**Primer**	**Position**	**Sequence (5′->3′)**
A1	A1F	−232	CCCGGTTGGACTAGGAAATGATGGGCTT
	A1R	6442	GTCCTGGTACTGGTTTGGGGGCTCTTCACAG
A2	A1F	−232	CCCGGTTGGACTAGGAAATGATGGGCTT
	A2R	2062	CACTAACCTGCACCACCACCCC
A3	A3F	1588	GGCGAGGCAGCAGGATTACAGG
	A3R	5002	CCGCACGCCACCTTCCCAATAA
A4	A4F	4525	CACCTTCCTGGGCTCGCTCACG
	A1R	6442	GTCCTGGTACTGGTTTGGGGGCTCTTCACAG


## Results

### Cloning of arylsulfatase I cDNA

By the 5′-end single-pass sequencing analysis of 24,000 clones isolated from ARPE-19 cDNA libraries, we identified one full-length cDNA clone each for *ARSI* and *SUMF1* [16]. To obtain *ARSA* and *ARSB* cDNA clones, PCR-based screening was performed against the remaining 69,504 clones. As a result, one full-length cDNA clone was identified for *ARSA*, but not for *ARSB*. Table 5 shows the size of the isolated full-length cDNA clones, the size of the protein encoded by these cDNAs, and the length of the 5′ untranslated region (UTR) and 3′ UTR with the corresponding RefSeq data. Every clone had an additional G at the 5′ end, which is a characteristic of a full-length cDNA clone prepared using the vector-capping method [15]. The length of the 5′ UTR was somewhat different between the obtained cDNA and RefSeq due to the diversity of the transcriptional start sites. The coding region of the *ARSI* and *ARSA* cDNAs had the same nucleotide sequence as the corresponding RefSeq, but there was one synonymous substitution, Thr(ACT)372Thr(ACC), in the coding region of the *SUMF1* cDNA clone. Based on comparison with the genome sequence, two sequence variations were found at the 3′ UTR of the *ARSI* cDNA clone, and ten sequence variations were found at the 3′ UTR of the *SUMF1* cDNA clone.

**Table 5:** Full-length cDNA clones isolated in this study.

**Gene**	**GeneID**	**HP number**	**Clone name**	**Accession number**	**mRNA (bp)**	**5′ UTR (bp)**	**Protein (aa)**	**3′ UTR (bp)**
*ARSI*	340075	HP10847	ARe01G09	AB448735	3159	580	569	869
			RefSeq	NM_001012301.2	3167	589	569	868
*ARSA*	607574	HP07238	ARiS019G20	AB448736	2014	374	507	116
			RefSeq	NM_000487.4	2053	406	507	123
*SUMF1*	285362	HP06735	ARiS078E09	AB448737	2127	8	374	994
			RefSeq	NM_182760.2	2148	25	374	998


Each cDNA clone was fully sequenced using a primer walking method. The sequence data was available from GenBank/EMBL/DDBJ under the corresponding accession number.

### Expression profile of arylsulfatase I mRNA

**Figure 1:**
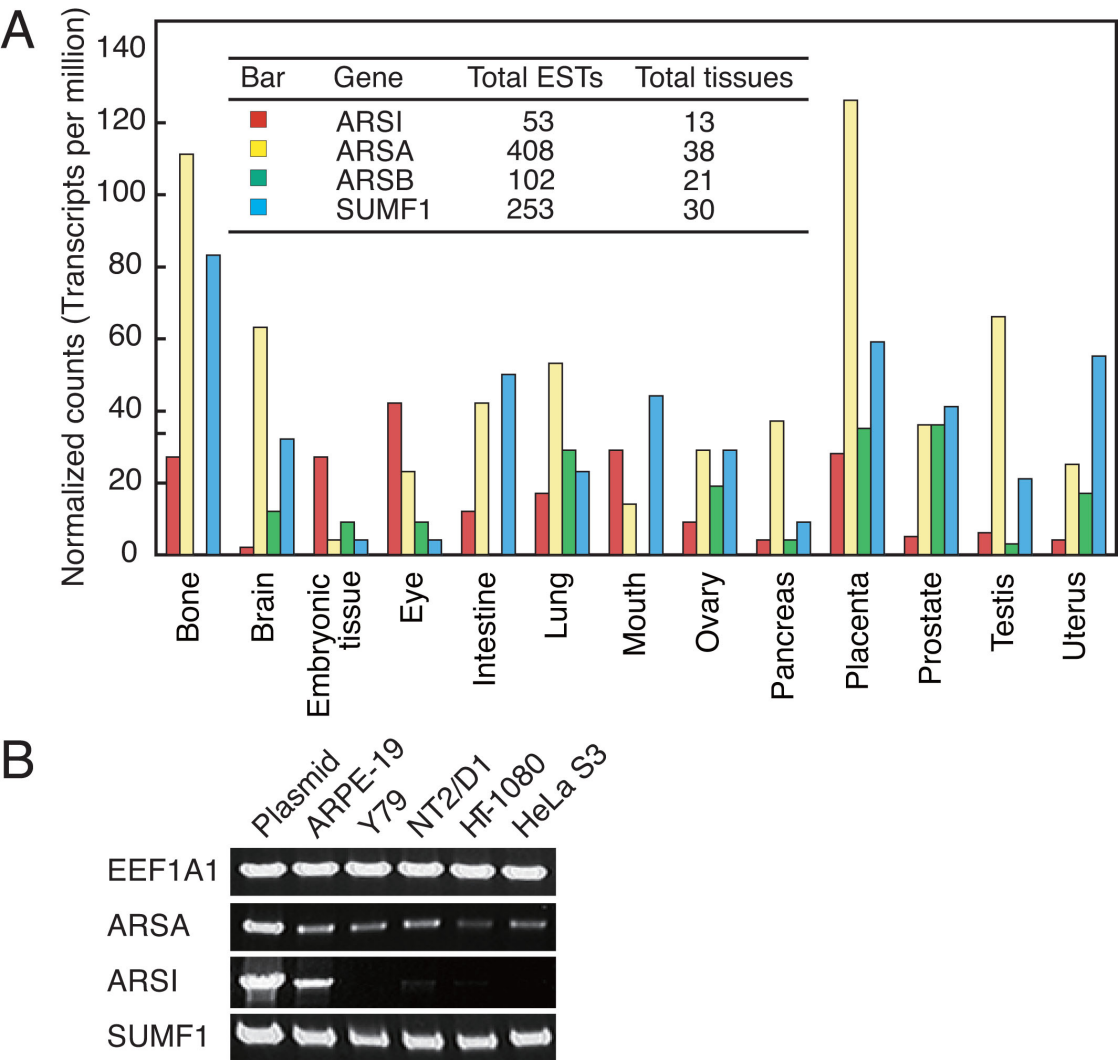
Expression profile of arylsulfatase-related genes. **A:** Thirteen tissues expressing *ARSI* were described based on the expressed sequence tag (EST) counts of UniGene database. The total EST counts and the total number of tissues expressing each gene are listed in the inset table. **B:** Arylsulfatase-related gene expressions in human cell lines were analyzed using RT–PCR. A eukaryotic elongation factor 1 alpha 1 (*EEF1A1*) gene was used as an internal control.

The expression profiles of *ARSI*, *ARSA*, *ARSB*, and *SUMF1* were obtained based on the UniGene EST count data as shown in Figure 1A. *ARSA* was expressed in 38 of 45 tissues and its EST count was highest among the four genes. In contrast, *ARSI* was expressed in only 13 tissues, and the EST count was one order of magnitude lower than that of *ARSA*. To obtain a more accurate expression profile for *ARSI*, we rechecked the EST database with BLAST. Hit sequences were clustered considering the presence of 5′-end and 3′-end ESTs derived from the same clone. As a result, the tissues expressing *ARSI* were, in order from the top, placenta (7 ESTs), retinoic acid-treated embryonic stem cell (6), retinoic acid-treated NT2 embryonic carcinoma cell line (3), colon tumor (3), fetal eyes (3), and lens (3), suggesting that *ARSI* is preferentially expressed in placenta, embryonic cell, and eye. These results are consistent with the previous report based on northern blot analysis that *ARSI* is detected only in placenta and fetal tissues [6]. It should be noted that the EST count of *ARSI* was larger than that of *ARSA* or *SUMF1* in embryonic cell and eye.

Figure 1B shows the results of RT–PCR performed using human RPE cell line ARPE-19, human retinoblastoma Y79, and other cell lines. The *ARSI* mRNA was detected in ARPE-19 but not in Y79, suggesting that RPE is one of the cells preferentially expressing *ARSI*. NT2/D1 and HT-1080 showed a faint band.

### Subcellular localization of arylsulfatase I

**Figure 2:**
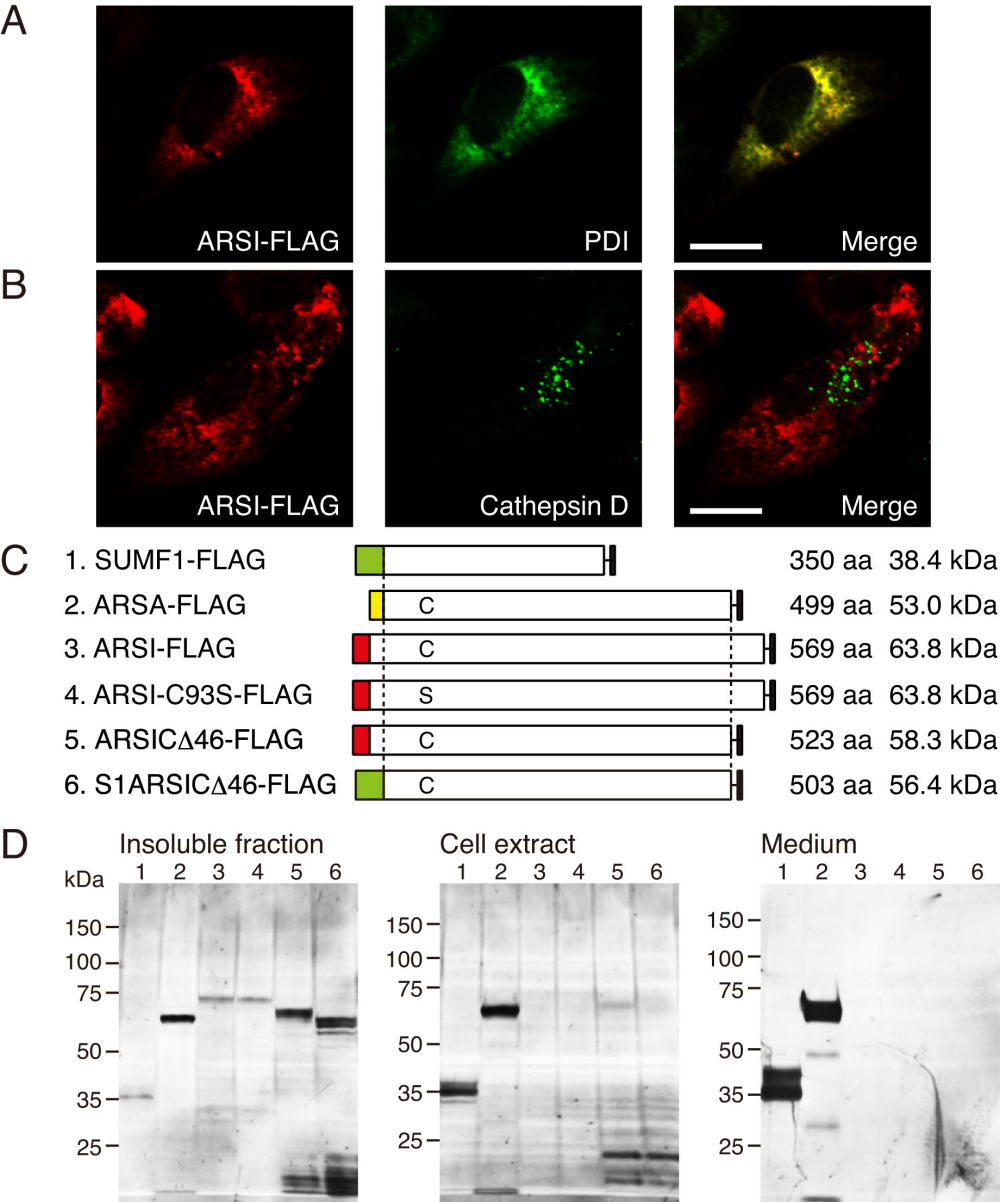
Transient expression of arylsulfatase-related genes. **A** and **B:** Endogenous PDI (ER marker, **A**) and cathepsin D (lysosome marker, **B**) were immunostained. ARPE-19 cells were transfected with 6 μg of the expression vector ARSI-FLAG. Scale bars, 10 μm. **C:** The structures of FLAG-tagged protein constructs were depicted. The colored box at the N-terminal represents a signal sequence. The active site of ARSI, Cys (C), was replaced by Ser (S) in a mutant derivative. The size of each secreted mature protein was represented. **D:** The expression products were analyzed with western blot. ARPE-19 cells were transfected with 1 μg SUMF1-FLAG, 5 μg ARSA-FLAG, or 8 μg ARSI derivatives, and cultured in serum-containing media for 22 h and then in serum-free media for 9 h. Loaded were 20% of the total sample for insoluble fraction, 10% for cell extract, and 25% for medium. The lane number corresponds to the construct number of the FLAG-tagged protein described in **C.**

To examine the subcellular localization of ARSI, we transfected the expression vector of FLAG-tagged *ARSI* (*ARSI-FLAG*) into ARPE-19 cells. The ER was detected using anti-PDI antibody, and the lysosomes were detected using anti-cathepsin D antibody. Indirect immunofluorescence staining and confocal microscopy showed that ARSI-FLAG was localized in the intracellular granular structures and colocalized with the ER (Figure 2A) but not with the lysosomes (Figure 2B). ARSI-FLAG also was colocalized with the coexpressed SUMF1-myc, and the distribution pattern of ARSI-FLAG was similar regardless of the coexpression of *SUMF1-myc* (data not shown). ARSI possesses additional 47- and 45-amino acid residues at the carboxyl-terminal compared to ARSA and ARSB, respectively. The deletion of the C-terminal residues did not affect the distribution pattern of the expression product (data not shown).

### Production of FLAG-tagged proteins

To characterize the expression products of SUMF1 and ARSs, we constructed the expression vectors of FLAG-tagged genes (*SUMF1-FLAG*, *ARSA-FLAG*, *ARSI-FLAG*, and *ARSI-C93S-FLAG*) as shown in Figure 2C (constructs 1–4). *ARSI-C93S-FLAG* was constructed as a mutant derivative by replacing a putative active site of ARSI, Cys at position 93, with Ser. Each fraction of the cultured cells transfected by these expression vectors was analyzed by SDS–PAGE and western blotting. As the production levels of SUMF1-FLAG were larger than those of the ARSs, a smaller amount of expression vector for *SUMF1-FLAG* was used for transfection. As shown in Figure 2D, ARSA-FLAG and SUMF1-FLAG were detected in all fractions, including the medium, but ARSI-FLAG was visible only in the insoluble fraction under these conditions. The size of ARSA-FLAG was 63.5 kDa, which is in agreement with the size estimated from the sum of glycosylated ARSA (62 kDa) [18] and the FLAG tag (1.2 kDa). The smaller three bands seemed to be degradation products. SUMF1-FLAG was observed as two bands of 36 kDa and 39 kDa in the cellular extracts, and two bands of 36 kDa and 41 kDa in the medium. The size of ARSI-FLAG detected in the insoluble fraction was 72 kDa, which was larger than the calculated value 63.8 kDa based on its amino acid sequence. This result suggests that the product was glycosylated through four potential N-glycosylation sites (Asn276, Asn288, Asn466, and Asn496) in ARSI. The size and production levels of ARSI-C93S-FLAG were the same as those of wild-type ARSI-FLAG.

### Production of ARSI derivatives

As wild-type ARSI could not give a large amount of expression products, we designed two derivatives, ARSICΔ46-FLAG and S1ARSICΔ46-FLAG (Figure 2C, constructs 5 and 6). ARSICΔ46-FLAG was constructed by deleting the C-terminal 46 amino acids that lacks in ARSA and ARSB. S1ARSICΔ46-FLAG was prepared based on ARSICΔ46-FLAG by replacing the signal sequence of ARSI with that of SUMF1, because the signal peptide of SUMF1 was likely to be involved in high production levels of SUMF1-FLAG. ARSICΔ46-FLAG and S1ARSICΔ46-FLAG were detected in the insoluble factions at positions of lower molecular mass, as expected from the decrease caused by the deletion or replacement of amino acid residues. ARSICΔ46-FLAG gave a weak band in the cell extract. The production levels of ARSI-FLAG were one-third those of ARSA-FLAG in the insoluble fraction. The C-terminal deletion increased the production levels to twice those of ARSI-FLAG. However, the replacement of the N-terminal signal sequence of ARSICΔ46 by that of SUMF1 did not induce a significant increase in the production levels.

### Arylsulfatase activity of expression products in cellular fractions

**Figure 3:**
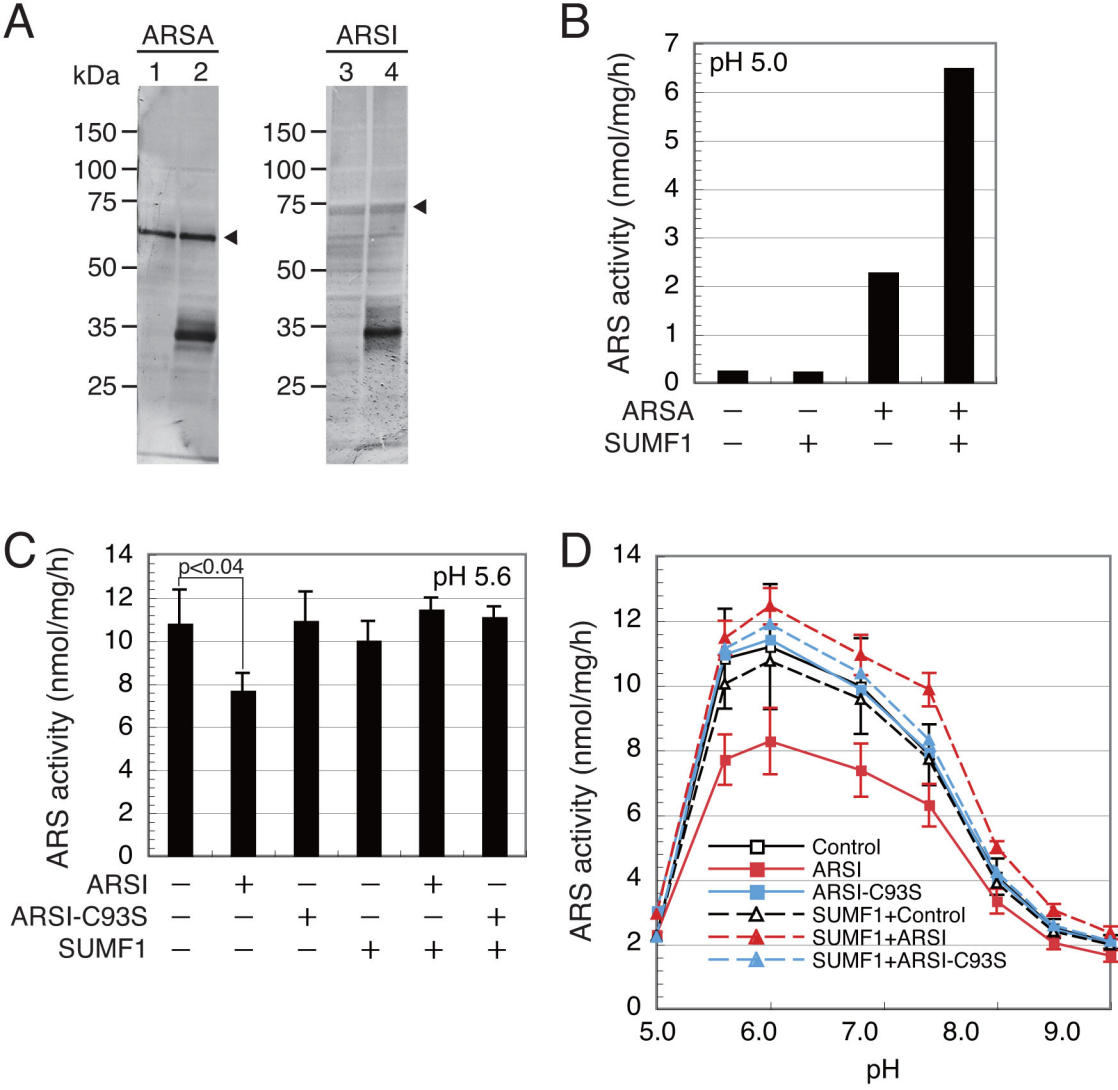
Arylsulfatase activity of cellular extracts. **A:** Cellular extracts were analyzed with western blot. Transfected were 5 μg *ARSA-FLAG* (lanes 1 and 2), 8 μg *ARSI-FLAG* (lanes 3 and 4), and 1 μg *SUMF1-FLAG* (lanes 2 and 4) into ARPE-19 cells .The transfected cells were cultured in media for 72 h. The amount of the loaded sample was 10 μg protein for ARSA and 12.5 μg protein for ARSI. **B:** The ARS activity of ARSA-FLAG was measured at pH 5.0 using 0.5 mM 4-MUS as a substrate. **C:** The ARS activity of ARSI-FLAG was measured at pH 5.6, using 5 mM 4-MUS as a substrate. The activity was calculated as the value per mg of cellular protein in each fraction. **D:** The pH dependence of ARS activities was shown. The ARS activities in **B, C,** and **D** give the means of three independent experiments. Bars indicate standard deviations. The error bars in **B** were omitted because the differences between samples were obvious.

As the production of ARSI-FLAG was confirmed, the ARS activity was measured using the cellular extracts. Although we did not observe any band in the cellular extracts under the aforedescribed culture conditions, we could detect a weak but clear band of ARSI-FLAG by changing the culture conditions as shown in Figure 3A. As SUMF1 is known to activate ARSs, *SUMF1-FLAG* was coexpressed for supplementation of SUMF1. Figure 3A shows that every expression product was observed in the cellular extracts. Although the ratio of transfected expression vectors (*ARSA-FLAG*, *ARSI-FLAG*, and *SUMF1-FLAG*) was 5:8:1, the production levels of ARSI-FLAG were significantly low.

First, the activity of ARSA-FLAG was measured using 4-MUS as a substrate. The cellular extract of *ARSA-FLAG*-expressed cells showed markedly high ARS activity compared with the endogenous activity (Figure 3B). Coexpression of *SUMF1-FLAG* increased the ARS activity, indicating that some of the overproduced ARSA-FLAG was activated by endogenous SUMF1 and that the remaining excess ARSA-FLAG was further activated by the coexpressed *SUMF1-FLAG* product.

The ARS activity of the cellular extract of *ARSI-FLAG*-expressed cells was as low as the endogenous activity (Figure 3C). As shown in Figure 3B, the endogenous ARS activity was >10-fold lower than that of *ARSA-FLAG*-expressed cells. Thus, a 10-fold higher substrate concentration, 5 mM, was used to detect a small difference in activity. The optimum pH of the endogenous ARS activity was around 6 (Figure 3D). Unexpectedly, the expression of *ARSI-FLAG* caused a decrease in endogenous ARS activity. The coexpression with *SUMF1-FLAG* resulted in a recovery of and even slight increases in activity at neutral pH range. In contrast, the expression of *ARSI-C93S-FLAG* did not induce such a decrease in endogenous ARS activity, and coexpression with *SUMF1-FLAG* did not change this activity. These results suggest that the overexpressed ARSI-FLAG in the cell extracts had no ARS activity and caused the decrease in endogenous ARS activity by a mechanism related to the activation of ARSI with SUMF1.

### Arylsulfatase activity of expression products secreted into media

**Figure 4:**
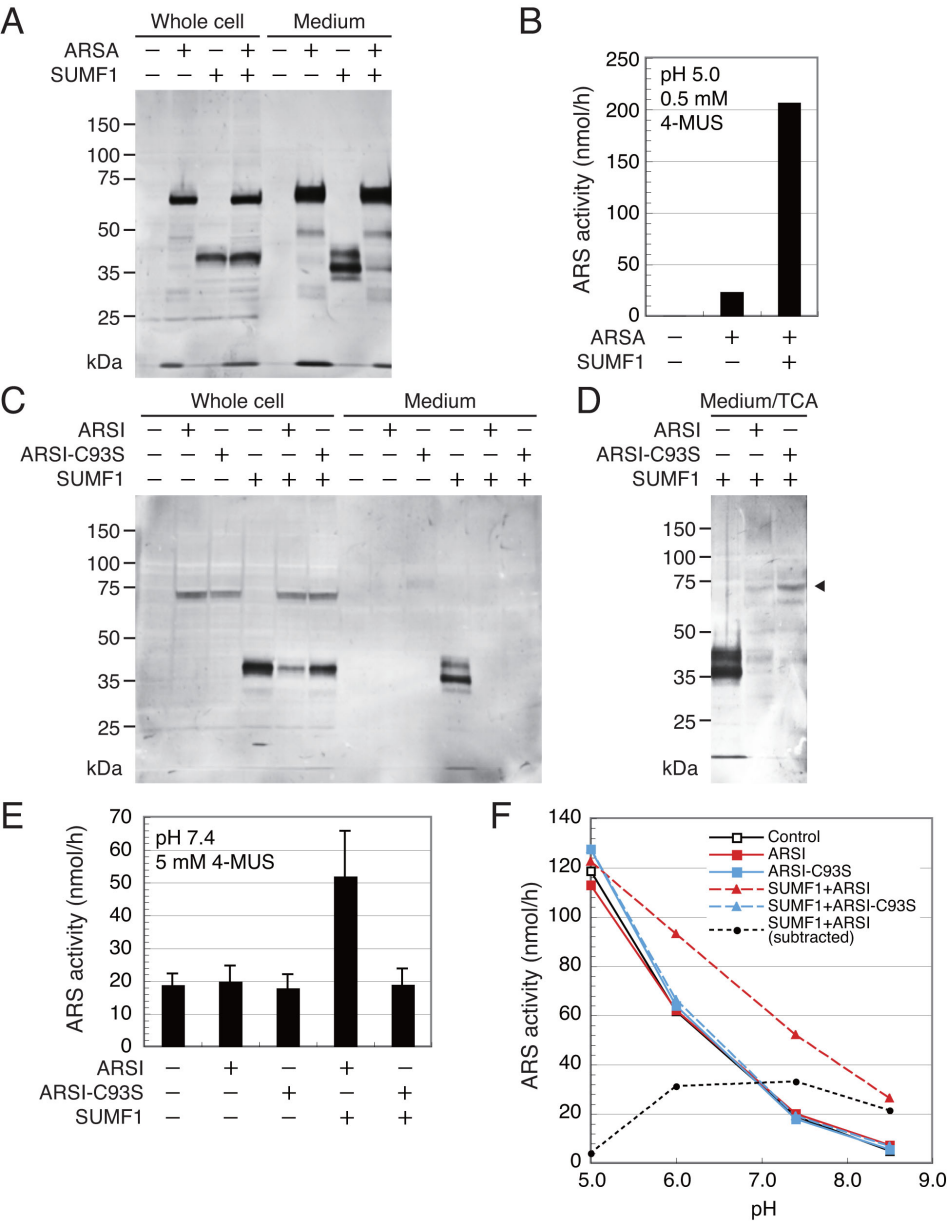
Arylsulfatase activity of media. **A:** Arylsulfatase A (ARSA) products were analyzed with western blot. ARPE-19 cells were transfected with 5 μg *ARSA-FLAG* and 1 μg *SUMF1-FLAG*, and cultured in serum-containing media for 2 h and then in serum-free media for 22 h. Loaded was 12.5% of the total sample. **B:** The ARS activity of ARSA-FLAG was measured using 0.4% of the total medium. **C:** Arylsulfatase I (ARSI) products were analyzed with western blot. Transfected were 8 μg ARSI-FLAG, 8 μg *ARSI-C93S-FLAG*, and 1 μg *SUMF1-FLAG* into ARPE-19 cells. **D:** The trichloroacetic acid (TCA)-induced precipitate of the medium was analyzed with western blot. **E:** The ARS activity of *ARSI-FLAG* was measured using 20% of the total medium. **F:** The pH dependence of ARS activities was shown. The ARS activities in **B, E,** and **F** give the means of three independent experiments. Bars indicate standard deviations.

The overexpression product of *ARSA-FLAG* or *SUMF1-FLAG* was secreted into the medium as shown in Figure 2D. *ARSA-FLAG* was introduced into ARPE-19 cells with or without *SUMF1-FLAG*, and the products in the cellular fractions and media were analyzed by SDS–PAGE and western blotting. Interestingly, the band of SUMF1-FLAG in the medium almost disappeared when there was coexpression with *ARSA-FLAG* (Figure 4A). The high activity of secreted ARSA-FLAG was detected at acidic pH, and the coexpression with *SUMF1-FLAG* drastically increased the ARSA activity (Figure 4B). These results suggest that the endogenous SUMF1 is not enough to activate the overproduced ARSA-FLAG.

The band of ARSI-FLAG was detected in the cellular fraction, but not in the concentrated medium (Figure 4C). During the concentration process of the conditioned medium, the degradation of the secreted ARSI may occur. When the medium was treated with TCA without the concentration process, the precipitate showed the band of ARSI-FLAG (Figure 4D), suggesting that ARSI-FLAG was secreted into the medium and that its amount in the concentrated medium was too small to be detected. As in the case of ARSA-FLAG, the band of SUMF1-FLAG in the medium disappeared when it was coexpressed with *ARSI-FLAG* or *ARSI-C93S-FLAG*. It should be noted that the band of SUMF1 in the cellular fraction also was reduced by coexpression with *ARSI-FLAG*.

The ARS activity of the conditioned medium of *ARSI-FLAG*-expressed cells was as low as that of the endogenous secreted ARS at every pH (Figures 4E,F). As shown in Figure 4B, the endogenous ARS activity was approximately 100 fold lower than the medium ARS activity of *ARSA-FLAG*-expressed cells. The optimum pH of endogenous ARS activity was acidic, suggesting that this activity may result from secreted lysosomal ARSs such as ARSA [19]. The difference in optimum pH between the endogenous ARS activities of the cell extracts (Figure 3D) and the medium (Figure 4F) may reflect the difference in endogenous ARS species. Only when *ARSI-FLAG* and *SUMF1-FLAG* were coexpressed did the media show significant ARS activity—2.5 fold higher than the endogenous activity at pH 7.4 (Figure 4E). The ARS activity of the secreted ARSI-FLAG was estimated by subtracting the endogenous activity (Figure 4F). The optimum pH of the secreted ARSI-FLAG seemed to be around pH 7. However, ARSI-C93S-FLAG did not show any activity. These results indicate that ARSI is activated by SUMF1 through modification of Cys93 into FGly, is secreted into the medium, and shows ARS activity at neutral pH.

### Sequence variation of arylsulfatase I gene locus

Patients with some forms of MPS that were caused by the mutation of *ARS*s are known to show symptoms characteristic of RP [8-13]. Thus, *ARSI* preferentially expressed in RPE can be a candidate gene responsible for RP. We have previously performed mutation screening of rhodopsin gene in Japanese RP patients. The same genome DNA sets isolated from 68 RP patients were used for mutation screening of the *ARSI* gene locus. The *ARSI* gene is located at chromosome 5q33.1 and is composed of two exons spanning a 6.4 kb region. The entire region was amplified by PCR and fully sequenced. Table 6 shows a total of 25 sequence variations, including ones found in this study and ones registered in the single nucleotide polymorphism database (dbSNP). Our samples contained 14 single nucleotide substitutions, seven of which were not registered in dbSNP. Out of seven novel single nucleotide substitutions, four were rare substitutions, and three (SV10, SV22, and SV25) were SNPs with >8% minor allele frequency (MAF). However, 11 substitutions registered in dbSNP were not found in our samples. Two synonymous substitutions (SV19 and SV22) were found in the coding region, but there was no nucleotide sequence variation causing an amino acid substitution. The analysis of the control subjects was not performed because no candidate mutation was found.

**Table 6:** Nucleotide sequence variations observed in the arylsulfatase I gene locus.

**Marker name**	**SNP name**	**Region**	**Amplicon**	**Position**	**Alleles major/minor**	**dbSNP ID**	**Minor allele frequency(%)**
SV01	SNP01	Exon 1	A2	−272	C/T	rs968431	30.15
SV02	-	Intron	A2	503	T/G	-	0.74
SV03	SNP02	Intron	A2, A3	1128	A/T	rs10463232	2.21
SV04	-	Intron	A3	1283	C/T	rs930209	0.00
SV05	-	Intron	A3	1314	G/A	rs7445044	0.00
SV06	-	Intron	A3	1328	G/T	-	0.74
SV07	SNP03	Intron	A3	1345	C/T	rs930210	12.50
SV08	-	Intron	A3	1440	G/A	rs11956540	0.00
SV09	-	Intron	A3	1678	C/T	rs4286653	0.00
SV10	SNP04	Intron	A3	1733	T/C	-	12.50
SV11	-	Intron	A3	1769	G/A	rs11956439	0.00
SV12	SNP05	Intron	A3	2293	C/A	rs12522210	9.56
SV13	-	Intron	A3	2318	A/-	rs34043807	0.00
SV14	SNP06	Intron	A3	2674	G/A	rs9324643	13.97
SV15	-	Intron	A3	2991–2995	ACGAG/-	-	0.74
SV16	-	Intron	A3	3534	C/T	rs11744744	0.00
SV17	SNP07	Intron	A3	3612	C/T	rs6579786	9.56
SV18	SNP08	Intron	A3	3641	C/T	rs6579785	22.06
SV19	-	Exon 2	A3	4005	C/T (Gly185Gly)	-	0.74
SV20	-	Exon 2	A3, A4	4086	C/T (Tyr212Tyr)	rs6579784	0.00
SV21	-	Exon 2	A4	4225–4226	-/C (frame shift)	rs34775469	0.00
SV22	SNP09	Exon 2	A4	4344	T/C (Asn298Asn)	-	8.09
SV23	-	Exon 2	A4	5466–5467	-/C	rs34570494	0.00
SV24	-	Exon 2	A4	5640	C/A	rs17111118	0.00
SV25	SNP10	Exon 2	A4	5644	C/T	-	11.76

Position 1 corresponds to the nucleotide A of the initiation codon of the ARSI gene. Sixty-eight unrelated patients with retinitis pigmentosa were subjected to mutation screening. The sequence variations include ones found in this study and ones registered in the single nucleotide polymorphism database (dbSNP). The sequence variation with >1% minor allele frequency was defined as SNP.

## Discussion

This study arose from the discovery of the *ARSI* cDNA clone during the comprehensive analysis of genes expressed in the RPE cell line ARPE-19 [15,16]. Expression profile analysis using several cell lines showed that *ARSI* is preferentially expressed in ARPE-19 but not in Y79. As the mutation of *ARS*s has been known to cause lysosomal storage diseases accompanying RP-like symptoms, we thought that *ARSI* preferentially expressed in RPE could be a candidate gene responsible for RP. We have previously performed mutation screening of the *RHO* gene locus using the genome DNA of 68 RP patients [17]. The same samples were used for mutation screening of the *ARSI* gene locus. Unfortunately, we found no missense or nonsense mutations but two synonymous substitutions, suggesting that *ARSI* is not a causative gene for the patients examined in this study. The sequence variation registered in dbSNP, SV21 (rs34775469, Table 6), is located in the coding region and causes a frame shift by insertion of one nucleotide. This shift is expected to produce C-terminal truncated ARSI. However, there is no available information as to whether subjects possessing this variation suffer from visual impairment.

During our mutation screening of the *ARSI* gene locus, the identification of *ARSI* as *ARSB* paralog [5] and the cloning and expression of the *ARSI* cDNA [6] were reported. Obaya examined the expression of *ARSI-FLAG* in HeLa cells and observed the band of ARSI-FLAG by SDS–PAGE and western blotting, but failed to show clear subcellular localization and its ARS activity [6]. To characterize the ARSI protein, we tried to overexpress the *ARSI* cDNA and to determine the ARS activity of ARSI. As ARSs are known to require activation by SUMF1 in the ER, we obtained full-length cDNA clones encoding SUMF1 and ARSA from the ARPE-19 cDNA library. Using these clones, we examined the transient expression of these cDNAs in ARPE-19 cells. ARSI-FLAG was shown to colocalize with the ER marker but not with the lysosomal marker, suggesting that the overproduced ARSI exists in the ER. SDS–PAGE analysis showed that the band of the expression product of *ARSI-FLAG* was observed in the cellular extracts and the insoluble cellular fraction, but that their production levels were lower than those of ARSA-FLAG or SUMF1-FLAG. Most of the overproduced ARSA-FLAG and SUMF1-FLAG were secreted into the media, whereas a visible band of ARSI-FLAG could not be observed in the concentrated media showing the ARS activity. The only difference among these expression vectors is the nucleotide sequence of the coding region. The replacement of the signal sequence by that of SUMF1 and the deletion of the C-terminal extension little improved the production levels of ARSI-FLAG. One possible explanation for the low production of ARSI is that ARSI is unstable and easily degraded in the ER or extracellular space. To obtain a large amount of ARSI protein, further investigation is required.

During expression analyses of ARS-related genes, we found interesting phenomena indicating that most of the SUMF1-FLAG was secreted into medium by the sole expression of *SUMF1-FLAG*, but not by coexpression with *ARSA-FLAG* or *ARSI-FLAG*. There are three possible explanations for this phenomenon: 1) the decrease in expression levels due to competition with the expression of *ARS*s; 2) the intracellular retention of the product; and 3) the degradation of the product. As the production levels of ARSA-FLAG did not change, the first explanation may not be appropriate. As the amount of the intracellular product did not change, or rather decreased, the possibility of the intracellular retention also may be negligible. In relation to this, we should note the differences between the intracellular (39 kDa) and secreted forms (41 kDa, 36 kDa, and 33 kDa) of SUMF1-FLAG (Figures 4A,C). Similar results have been reported regarding the difference between intra- and extracellular His-tagged SUMF1 [20], with the size heterogeneity being explained by the differences in glycosylation and N-terminal truncation. Consequently, our third explanation is most plausible. It has been reported that ARS activities are regulated by the interaction with SUMF1 and SUMF2 [21]. The present results led us to suppose that SUMF1 is degraded after interacting with ARSs. Further investigation of the regulation mechanism of ARSs by SUMF1 is necessary to confirm the present hypothesis.

The ARS activity of ARSI-FLAG could not be detected in the cellular extract that showed a visible band of expression product, but the conditioned media of cells coexpressed with *SUMF1-FLAG* showed significant ARS activity, despite the absence of the visible band of ARSI-FLAG. Furthermore, the overproduction of ARSI-FLAG, but not ARSI-C93S-FLAG, caused the decrease in endogenous ARS activity in the cell extracts. This decrease was recovered by coexpression with *SUMF1-FLAG*. All these phenomena could be explained by assuming the following: 1) ARSI is a secreted protein; 2) the secreted ARSI is rapidly degraded in the ER or medium; 3) when ARSI is overproduced, a part of overproduced product is retained in the ER as a denatured form; and 4) overproduction of ARSI causes degradation of SUMF1. The activity of ARSI-FLAG in the medium may result from a low amount of ARSI-FLAG escaping degradation. Expectedly, the TCA-precipitated product of the conditioned medium gave the band of ARSI-FLAG that could not be detected in the concentrated medium. The decrease in endogenous ARS activity in the cell extract may be caused by degradation of endogenous SUMF1 due to overproduction of ARSI-FLAG, and the endogenous ARS of expression vectors-free cells may bear the remaining activity. Consequently, the key issue to be solved is the degradation mechanism of ARSI and SUMF1.

The conditioned media of *ARSI-C93S-FLAG*-expressed cells coexpressed with *SUMF1-FLAG* showed no ARS activity. This result confirms that the active site of ARSI is Cys93 and that it is activated by SUMF1 as well as other ARSs. The low activity in the present study may be due to the rapid degradation of the secreted ARSI-FLAG. The optimum pH of ARSI-FLAG secreted into the medium was around neutral range, while lysosomal ARSs, such as ARSA and ARSB, show the highest activity at acidic pH. These results strongly suggest that ARSI is secreted and functions in the extracellular environment.

The characteristics of ARSI revealed by the present investigation, the possibility of extracellular ARS preferentially expressed in embryonic tissues, are a reminder that extracellular matrix sulfatases have been shown to be involved in signal transduction during embryonic development [22]. For example, QSulf1, which is a cell-surface sulfatase found in quail embryos, has been noted to be active in Wnt signaling and embryonic development [23]. Its human orthologs, HSulf1 and HSulf2, have been isolated and confirmed to be extracellular heparin-degrading endosulfatases [24]. These reports and our present findings led us to suppose that ARSI also functions during developmental stages, whereas the meaning of the preferential expression in the eye is unknown. To understand the function of ARSI, it is necessary to determine its physiologic substrate. From a structural perspective, the C-terminal extended amino acid residues of ARSI may play an important role in substrate recognition. The HSulf sulfatases and HGlcN6S, which catalyze the hydrolysis of glucosamine 6S residues, contain the C-terminal stretch of amino acids that exhibit high homology with a region of the GlcNAc transferase from *Arabidopsis thaliana*, suggesting that this region may be involved in substrate recognition [24]. The search for a target molecule interacting with the C-terminal residues of ARSI should provide information regarding its true substrate.

In conclusion, we succeeded for the first time in detecting the ARS activity of ARSI and found that ARSI may be secreted and function in the extracellular space. Further investigation will shed light on the role of ARSI in the function of RPE cells. ARSI seems not to be a lysosomal ARS, and thus to not be a causative gene in lysosomal storage disorders. However, *ARSI* preferentially expressed in the eye would still be a candidate gene causing inherited eye diseases for future mutation screening.
